# A Multidisciplinary Approach toward CMOS Capacitive Sensor Array for Droplet Analysis

**DOI:** 10.3390/mi15020232

**Published:** 2024-02-01

**Authors:** Hamed Osouli Tabrizi, Saghi Forouhi, Tayebeh Azadmousavi, Ebrahim Ghafar-Zadeh

**Affiliations:** 1 Biologically Inspired Sensors and Actuators, Department of Electrical Engineering and Computer Science, Lassonde School of Engineering, York University, Toronto, ON M3J 1P3, Canada; htabrizi@cse.yorku.ca (H.O.T.); sforouhi@yorku.ca (S.F.); 2 Department of Electrical Engineering, University of Bonab, Bonab 5551395133, Iran; tayebehazadmousavi@gmail.com; 3 Department of Biology, Faculty of Science, York University, Toronto, ON M3J 1P3, Canada

**Keywords:** CMOS, capacitive sensor, droplet analysis, life science applications, time of evaporation

## Abstract

This paper introduces an innovative method for the analysis of alcohol–water droplets on a CMOS capacitive sensor, leveraging the controlled thermal behavior of the droplets. Using this sensing method, the capacitive sensor measures the total time of evaporation (ToE), which can be influenced by the droplet volume, temperature, and chemical composition. We explored this sensing method by introducing binary mixtures of water and ethanol or methanol across a range of concentrations (0–100%, with 10% increments). The experimental results indicate that while the capacitive sensor is effective in measuring both the total ToE and dielectric properties, a higher dynamic range and resolution are observed in the former. Additionally, an array of sensing electrodes successfully monitors the droplet–sensor surface interaction. However practical considerations such as the creation of parasitic capacitance due to mismatch, arise from the large sensing area in the proposed capacitive sensors and other similar devices. In this paper, we discuss this non-ideality and propose a solution. Also, this paper showcases the benefits of utilizing a CMOS capacitive sensing method for accurately measuring ToE.

## 1. Introduction

In recent decades, biosensors have become integral to life science and biotechnology research. The current trend in the implementation of biosensors is toward creating point-of-care (PoC) testing and numerous parallel biorecognition arrays which make a major challenge of the monolithic integration of assays (biochemical, genomic, etc.) with biosensor arrays [1,2]. One widely used trend to address this challenge is incorporating complementary metal–oxide–semiconductor (CMOS) technology in the biosensor design (referred to as the CMOS biosensor), which provides the integration of a large number of transistors (i.e., enables array implementation) and cost-efficient and low-power consumption systems with a high production yield and robust functionality [3,4,5,6]. A CMOS-based biosensor comprises a microfluidic structure designed to direct samples toward sensing sites on the CMOS sensing chip. This chip integrates sensors and circuits to detect and transduce biological or chemical changes into electrical signals, which are then digitized and transferred to a computer for analysis. Microfabrication techniques are essential for developing microfluidics and integrating them onto the CMOS chip to ensure hermetic bonding between the chip and fluidic structure [7]. Despite the significance of microfluidics for various applications, this work primarily utilizes encapsulation methods for bonding and leaves the chip surface open for the introduction of the droplet. The main focus of this study is the development of a novel method for microelectronic sensing, incorporating a thermal-based capacitive CMOS sensor tailored for future life science applications.

Within the diverse applications of CMOS biosensors, there has been a notable focus on the extraction of droplet characteristics, providing essential information crucial for analytical purposes. Through the analysis of droplets, critical properties of their behavior on solid surfaces can be determined. These properties include the time of evaporation (ToE), evaporation rate (ER) [8], dielectric constant [9,10,11] humidity characteristics [12] thermal diffusivity [13], refractive index [14], and adhesion [15]. The field of CMOS biosensors has seen a multitude of techniques proposed for droplet analysis such as a magnetic sensor [16], nuclear magnetic resonance (NMR) sensor [17], optical sensor [14,18], thermal sensor [13], and capacitive sensor [19,20,21]. For instance a CMOS thermal sensor has been proposed by Cheng et al. [13] for the direct measurement of the diffusivity of liquid samples dropped onto the device. In another work presented by Saeidi et al. [21], a capacitive humidity sensor is designed, which exhibited capacitance with an almost linear relation to the relative humidity. Among these prospective technologies, the capacitive sensor array has been a prominent approach, offering high sensitivity and accuracy.

In this work, a novel droplet-sensing platform is introduced to monitor droplet evaporation, which is constructed of an array of 16 × 16 small electrodes (35 µm × 30 µm) with a capacitive interface circuit. This configuration enables the measurement of minute capacitance changes on the sensing electrodes when exposed to chemical solvents. While capacitive sensors offer advantages in detecting changes in dielectric properties, they encounter challenges in accurately discerning small dielectric changes during the evaporation of droplets, particularly in scenarios involving a low alcohol concentration in water–alcohol mixtures. This paper addresses the difficulties associated with using capacitive sensors for such applications and proposes a solution that focuses on analyzing the duration of the droplet presence before evaporation.

## 2. Related Works

For droplet analysis, the array of capacitive sensors offers a precise and sensitive means of detecting and measuring minute changes in capacitance, allowing for the accurate characterization and monitoring of droplet properties such as size, shape, and composition. Such an array can also be envisioned for PoC detection where the numerous parallel detectors enable multiplexing and result in reduced sample size, diagnosis time, cumbersomeness, and cost. On the other hand, the integration of microfluidic components with a sensor would enable the development of a complete lab on chip (LoC) microsystem, which introduces opportunities for affordable, energy-efficient, and portable systems, making it a viable solution for a wide range of applications, especially for PoC diagnostics [22,23,24,25]. Moreover, the combination of microfluidics with an array of sensors would, therefore, enable one to simultaneously measure the properties of different types of liquid.

**Figure 1:**
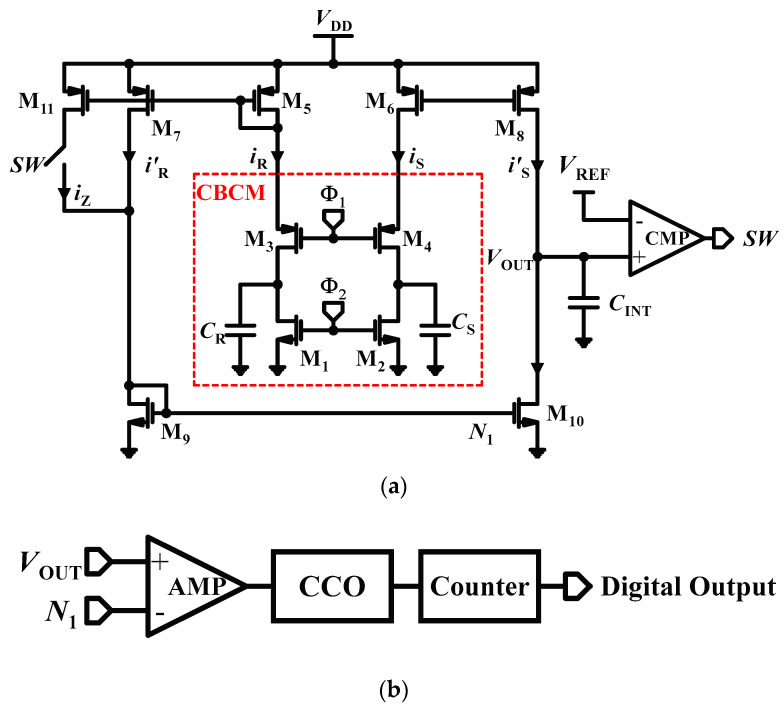
The core-CBCM capacitive sensor using: (**a**) ∑∆ structure; (**b**) CCO structure.

One of the widely used interface circuits to implement an array of sensors is known as a charge-based capacitance measurement (CBCM), which presents a compelling solution with its advantageous blend of high accuracy and low complexity, rendering it particularly well-suited for LoC applications [25,26]. The core CBCM is shown in Figure 1a (the part inside the dashed line). Two signal pulses (*Φ*_1_ and *Φ*_2_) are applied to two pairs of transistors (M_1,2_ and M_3,4_) to charge and discharge the reference capacitor (*C*_R_) and a sensing capacitor (*C*_S_) [27]. The difference between the capacitances of the *C*_R_ and *C*_S_ results in the instantaneous currents *i*_S_ and *i*_R_ flowing through the CBCM core branches, which are proportional to a variation in the analyte. The subtraction of these currents can be achieved by transistors of M_5–10_ as shown in Figure 1a [28]. Injecting the current into the integrating capacitance *C*_INT_ results in a voltage in which a linear relation between *V*_OUT_ and the input capacitance changes Δ*C* will be exited. The DC value can be converted to digital data through ΣΔ architecture as shown in Figure 1a. This structure of CBCM operated in voltage mode, which limited the dynamic range and caused integration to occur in the analog domain. Furthermore, this approach restricted the swing of the integrating capacitor voltage due to limitations in the supply voltage. To address this issue, a current-mode circuit was introduced in [29] specifically designed for low-supply voltage CMOS technologies, and its main concept is shown in Figure 1b. This circuit featured a core-CBCM capacitance-to-frequency converter (CFC), allowing for an improved input dynamic range (IDR). In this method, the amplified currents from the CBCM core are subtracted and directed to a current-controlled oscillator (CCO) [30]. The CCO effectively modulates the currents of the CBCM block into a pulse frequency, and then a counter is utilized to obtain the average output frequency.

Within our group’s extensive research we have introduced a series of core-CBCM capacitive sensors explicitly developed for monitoring a spectrum of liquid samples characterized by diverse dielectric constants, encompassing water [31], ethanol [32], methanol [31,33], propanol [31], dichloromethane [31,33], and acetone [31,33]. Our previous work, detailed in [31], involves the introduction of a wide dynamic range core-CBCM capacitive-to-digital converter (CDC) featuring two electrodes. This design is marked by its versatility, enabling the sensor to precisely capture dynamic variations in droplets with a greater thickness during the evaporation process. A distinguishing feature of this sensor is its ability to operate effectively without necessitating the thin layer of the liquid sample typically required to cover the sensor surface. This capability enhances its applicability and reliability in diverse sensing scenarios.

This paper highlights the advantages of the CBCM capacitive sensor array implemented in CMOS technology, emphasizing its increased accuracy in analyzing micro-liter droplet solutions.

## 3. Proposed Droplet-on-Chip Sensor

In this section, after outlining some possible tasks and applications of a droplet-on-chip (DoC) platform, the design metrics to achieve those goals will be introduced. Then, the CMOS capacitive sensor used for the proof of concept will be described.

### 3.1. Multi-Task DoC Platforms

CMOS capacitive sensors can be used for the analyses of binary droplets, such as water–ethanol (W-Et) and water–methanol (W-Mt) mixtures. In this section three capabilities of capacitive sensors comprising dielectric sensing, measuring ToE, and monitoring the location or shape of the droplet to develop a multi-task DoC platform has been investigated.

#### 3.1.1. Dielectric Sensing

The prevalent use of capacitive sensors often revolves around measuring the dielectric properties of liquid samples. Assuming interdigitated electrodes (IDEs) as the sensing elements and considering negligible edge effects, the capacitance of an IDE (*C*_IDE_) is directly proportional to the permittivity of the solution near the electrode [34]. For a mixture of water and ethanol with a known volume fraction of water (*γ*_w_) and alcohol (*γ*_e_) the effective permittivity (*ε*_m_) can be estimated using the permittivity values of water (*ε*_w_) and ethanol (*ε*_e_) at room temperature based on the Kraszewski Law [35]. This estimation can be calculated as (*ε*_m_)^0.5^ = *γ*_w_ (*ε*_w_)^0.5^ + *γ*_e_ (*ε*_e_)^0.5^. Furthermore, the rational partial derivatives of IDE capacitance and mixture permittivity are equivalent, represented as *∂C_IDE_/C_IDE_* = *∂ε*_m_*/ε*_m_. Let us define the mean value of ∂*C*_IDE_/*C*_IDE_ as the capacitance change ratio (*CCR*). Considering *γ*_w_ + *γ*_e_ = 1 where each volume fraction varies between 0 and 1 the *CCR* can be derived for *γ*_e_ ranging from 0 to 1 or ethanol percentages from 0 to 100% in the mixture. Employing the earlier calculations, *CCR* = 0.02 ∗ Ln(*ε*_e_*/ε*_w_)^0.5^. Considering the permittivity values of water, ethanol, and methanol at room temperature as 78.2, 24.55, and 32.7, respectively, the resulting *CCR* for the W-Et (water–ethanol) or W-Mt (water–methanol) compositions remains below 1.2%.

#### 3.1.2. Time of Evaporation Measurement

**Figure 2:**
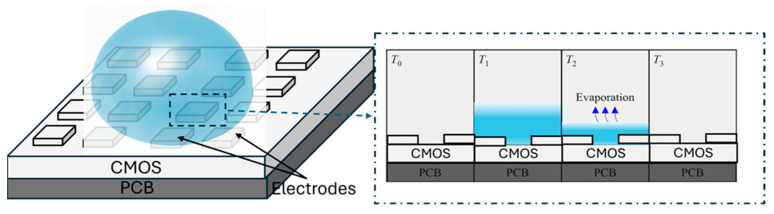
Droplet on capacitive sensor array: schematic of the droplet before (*T*_1_), during (*T*_2_, *T*_3_), and after evaporation (*T*_3_).

Figure 2 illustrate the principle of the proposed ToE sensing method. Once the sensing electrodes’ surface is exposed to the droplet the sensed capacitance is rapidly increased from the baseline, *C*_baseline_, in the dry-phase, *T*_0_, to the maximum value, *C*_max_. The volume of the droplet decreases due to evaporation until the thickness (*τ*) of the fluidic sample becomes lower than the specific length or so-called screen length (*SL*) of the sensor. During *T*_1_, we have *τ* > *SL* and the sensor is in its saturation region while during *T*_2_ we still have *τ* > *SL* and the sensor behavior can be seen in its saturation. When the layer of solution is lower than the SL, the output of the capacitive sensor varies until it becomes zero, which indicates the evaporation of the liquid (between *T*_2_ and *T*_3_). Therefore, the ToE is equal to the total time when *C*_max_ − *C*_baseline_ > 0. At *T*_3_ the droplet has undergone full evaporation.

Various numerical analysis methods, such as finite element analysis (FEA) and computational fluid dynamics (CFD) [36,37,38] have been employed by researchers to calculate the ToE and examine the evaporation of binary droplets experimentally [39,40,41,42,43]. Despite the ToE and evaporation rate being dependent on numerous parameters, under constant conditions, such as temperature and humidity, the average time of the evaporation change ratio (*TCR*) can be approximated as ∆ToE/ToE. This paper proposes the utilization of a capacitive sensor for measuring ToE.

#### 3.1.3. Monitoring the Shape and Location of Droplets

When employing an array of sensing electrodes, the identification of droplet location and shape atop the sensing area relies on the capability of underlying sensors to differentiate between covered and uncovered areas by the liquid. Despite µL-volume droplets occupying a significant space, the sensing area’s size is crucial for accurate droplet analyses. Larger electrodes, however, tend to have an increased offset capacitance, restricting the sensor’s input dynamic range and lowering the electrode capacitance ratio—i.e., the change in capacitance due to the sample relative to the offset capacitance without the sample. To address these challenges, employing a large array of small electrodes proves advantageous for the desired DoC platform. However, several factors influence the array’s design. Dense electrode configurations improve the shape, location, and volume estimation of the droplet, but pose challenges regarding crosstalk and noise when electrodes affect one another more profoundly in denser setups. Balancing these considerations is essential for optimal performance and accuracy in droplet analysis.

### 3.2. CMOS Capacitive Sensor Array

**Figure 3:**
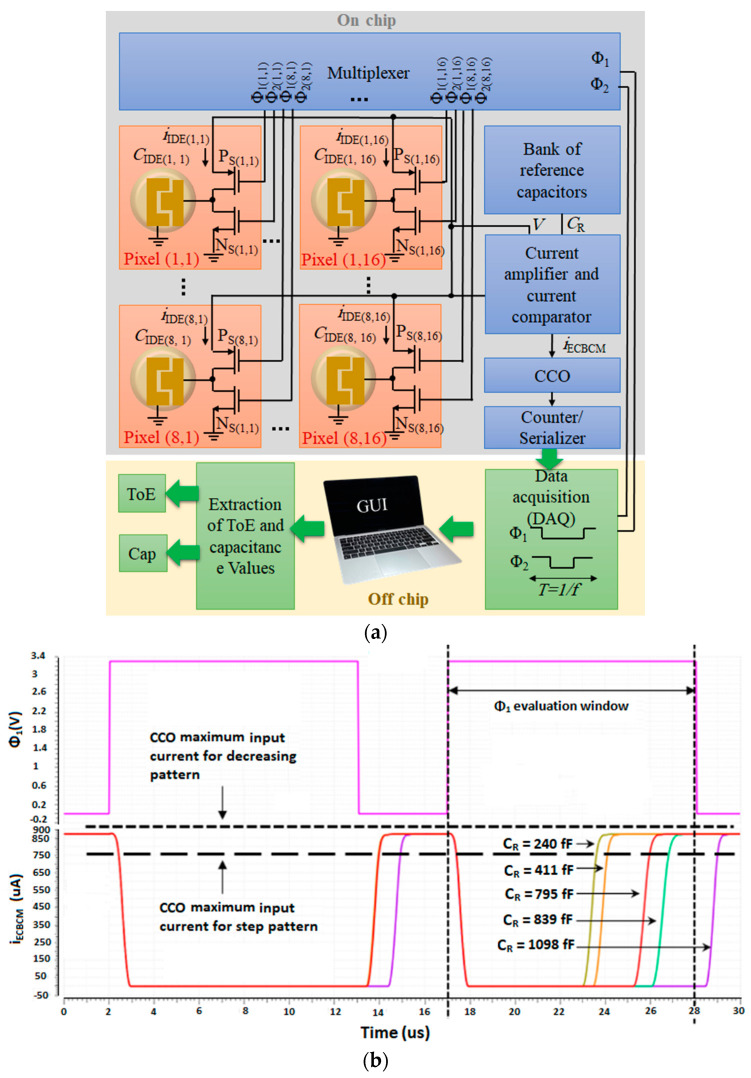
(**a**) An 8 × 16 compartment of the used core-CBCM capacitive array sensor; (**b**) The signals of Φ_1_, and *i*_ECBCM_ of the system shown in (**a**) for different reference capacitors (*C*_R_).

In our recent work [44], a CMOS capacitive array sensor that operates based on CBCM has been introduced. This sensor comprises two sets of 8 × 16 arrays, which are each linked to separate readout circuits. Unlike other reported multiplexed capacitive sensor arrays, such as in [34], the multiplexing technique employed in the array does not require additional switches in the current paths of the electrodes (see Figure 3a). In this circuit, each pixel located in the *i*th row and *j*th column, pixel(*i*,*j*), consists of an IDE and a pair of PMOS and NMOS switches, P_S_(*i*,*j*) and N_S_(*i*,*j*), controlled by two non-overlapping clock pulses, namely *Φ*_1_(*i*,*j*) or *Φ*_2_(*i*,*j*) with the same frequency *f*. *Φ*_1_(*i*,*j*) or *Φ*_2_(*i*,*j*) are used to select the IDEs and also play the role of the core of the CBCM method. At each time, only one of the pixels is turned on based on their *Φ*_1_(*i*,*j*) or *Φ*_2_(*i*,*j*). These pulses are generated using a multiplexer fed by two clock pulses, *Φ*_1_ or *Φ*_2_ produced by an off-chip microcontroller. When *Φ*_1_(*i*,*j*) or *Φ*_2_(*i*,*j*) of each pixel are low the capacitance of the corresponding IDE (IDE(*i*,*j*)) will be charged to a known voltage, *V*, and when the pulses become high, the capacitance will be discharged. It can be proved that the average of the capacitance current is proportional to the capacitance, $\bar{i_{IDE(i,j)}}=C_{IDE(i,j)}fV$. If we consider a similar structure with a reference capacitor, *C*_R_, which is insensitive to or separated from the analyte, instead of the sensing IDE which is sensitive to the analyte, it is possible to obtain a current, *i*_R_, whose average is proportional to *C*_R_ (or $\bar{i_{R}}\propto C_{R}$). The capacitance changes due to the presence of the analyte, ∆*C* = *C*_IDE(*i*_,*_j_*_)_ − *C*_R_ can be obtained by subtracting and averaging these two currents, $i_{IDE(i,j)}-i_{R}$.

Using an array of *C*_R_ values instead of a single *C*_R_ makes it possible to measure the capacitance in a wider dynamic range with high accuracy and without the need for calibration, as described in [20]. In this circuit, the difference between the *C*_S_ and all of the *N* values of *C*_R_ in a bank of capacitors is measured for each pixel. Three current mirrors and a current comparator were used to amplify and subtract the currents of the sensing and the reference capacitors, *i*_IDE(*i*,*j*)_ and *i*_R_, and generate a differential current called *i*_ECBCM_ = *i*_IDE(*i,j*)_ − *i*_R_. A CCO converts this current to some pulses. By counting these pulses using a counter/serializer, a digital output signal is generated which is the average of the differential current and proportional to ∆*C* = *C*_IDE(i,j)_ − *C*_R_.

Figure 3b illustrates the signals of *Φ*_1_ and the current *i*_ECBCM_ shown in Figure 3a. The evaluation window depicted in this figure demonstrates the interval during which the current, *i*_ECBCM_, is averaged or integrated. In an ideal case, the maximum value of *i*_ECBCM_ (or *i*_ECBCM,max_) should be lower than the IDR of the CCO. In other words, *i*_ECBCM,max_ < *I*_CCO,max_, where *I*_CCO,max_ is the maximum input current of the CCO by which it can oscillate. As seen in this figure, increasing *C*_R_ results in a shift in the current signal which means reducing ∆*C* = *C*_IDE(*i,j*)_ − *C*_R_. So when *i*_ECBCM,max_ < *I*_CCO,max_ the integral of *i*_ECBCM_ in the evaluation window will decrease by increasing *C*_R_. Consequently, the digital output will also follow a decreasing pattern with respect to the increasing *C*_R_.

But, if *i*_ECBCM,max_ > *I*_CCO,max_, we expect to see a step-like pattern because, in this case, as also shown in Figure 3b, the CCO oscillates only during the transition of *i*_ECBCM_ from low to high, and consequently, the current will only be integrated during this interval which determines the digital output. In this case, as long as the transition of current happens within the evaluation window, the output shows a higher digital output compared to when the transition happens outside of the evaluation window. In the sweep, as *C*_R_ keeps increasing, after some *C*_R_ value, no transition happens in the *Φ*_1_ evaluation window. This value is where we see the sharp drop in the step-like pattern. These two patterns will be demonstrated and discussed in Section 5.

## 4. Experimental Setup and Materials

This section is dedicated to describing chip fabrication and packaging, the testbench board, experimental setup, and sample preparation.

### 4.1. Chip Fabrication and Testbench

**Figure 4:**
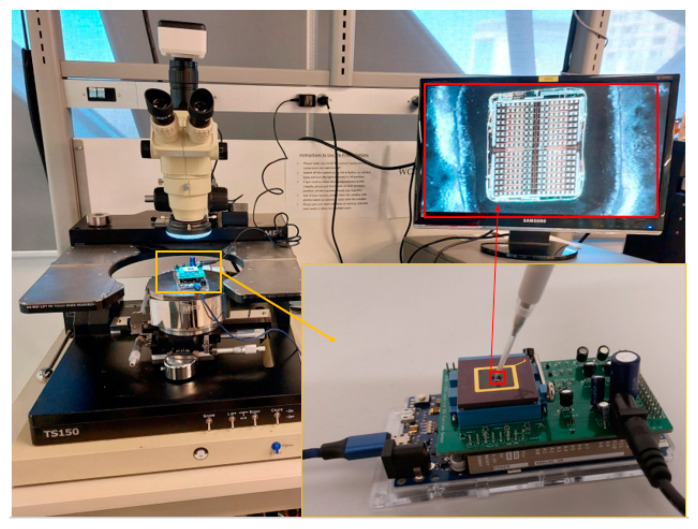
The testbench platform and sample introduction to the chip as well as the microscopic image of the passivated and non-passivated IDEs, and the entire experimental setup.

The CMOS capacitive sensor array used in this study was constructed using 0.35 µm CMOS technology and assembled within a commercial CPGA85 package, as reported in [33]. To safeguard the bonding wires from exposure to liquid, a UV-cured epoxy resin was employed for encapsulation. The electrodes were integrated onto the topmost metal layers (metal 4) of the technology. The chip consists of two identical compartments, each portrayed in Figure 4’s microscopic image containing 8 × 16 SiO_2_-passivated and 8 × 16 bare aluminum electrodes. Every electrode comprises 2 fingers, each 5 µm in length and width. The total sensing area measures 1326 × 1400 µm^2^, covering a space of 35 × 30 µm^2^. The chip features a programmable bank of capacitors enabling a sweeping range of 10 bits, encompassing values up to 1024 fF with a step size of 1 fF. A custom-designed PCB board was created to accommodate the new chip, incorporating five distinct voltage regulators and a multiplexer. Among the regulators, four were designated for providing analog and digital supply voltages (3.3 V), with two each for the right and left sides. The fifth regulator facilitated the necessary reference voltage (1.85 V) for the oscillator. To minimize noise interference, decoupling capacitors were strategically employed across input and output power supplies for all components. Additionally, an updated version of the graphical user interface (GUI) was developed specifically for data collection from 256 sensing electrodes. Clock pulse periods *Φ*_1_ and *Φ*_2_ were set at 15 µs. Furthermore precise pre-calibrated pipettes were utilized to extract a microliter volume of the sample and introduce it to the designated sensing areas.

### 4.2. Sample Preparation

For the evaluation of the concept and the capability of the capacitive sensor to measure the ToE, two types of binary droplets containing W-Et and W-Mt mixtures were employed. In each experiment, a specific volume of the sample containing *x*% of liquid 2 and (100 − *x*)% of liquid 1 is applied to the sensor where *x* is an integer ranging from 0 to 100 with steps of 10.

## 5. Results

In the following subsections, we demonstrate and discuss the simulation and experimental results of the sensor.

### 5.1. Characterization of the Sensor Array without Sample

**Figure 5:**
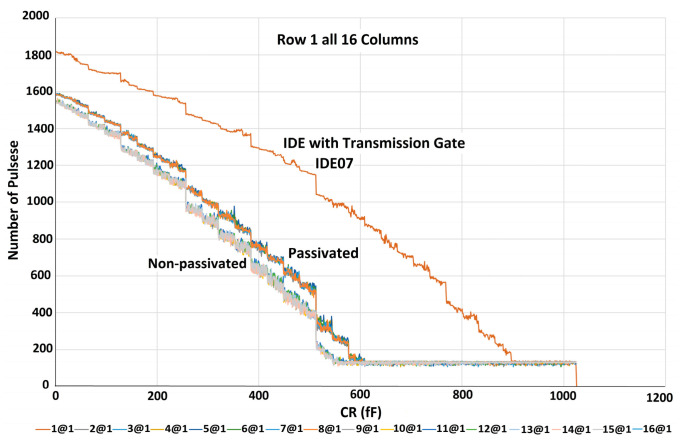
The decreasing pattern of the upper compartment.

**Figure 6:**
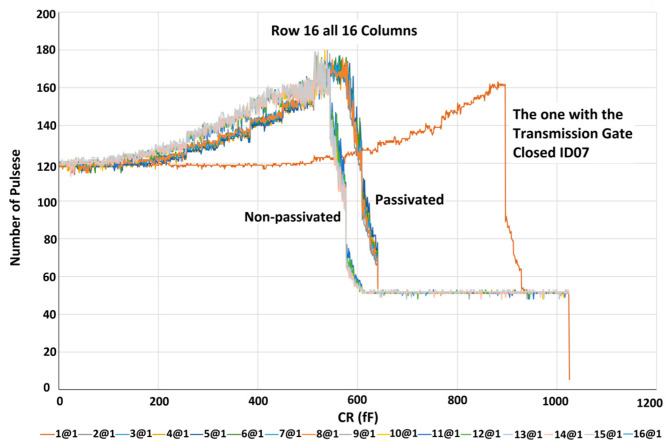
The step-like pattern of the lower compartment.

In the first step, before running the experiment in the presence of the sample, all 256 electrodes were scanned to obtain the baseline capacitance. For the characterization of the sensor, the digital outputs of all electrodes were measured while all the capacitors in the bank of capacitors were swept and were not exposed to any analyte. The experiments’ results clearly show the two different patterns discussed in Section 3.2 for the upper and lower compartments of some of the chips; however, the same layout has been copied for them. The decreasing pattern that has been shown in Figure 5 is an expected pattern for the ideal case which is due to a gradual decrease in ∆*C*. So an increase in *C*_R_ results in a gradual drop in the output of the chip creating a linear decreasing pattern. Figure 6 shows a step-like pattern.

To find the reason, we investigated the design by performing a corner analysis. Based on the simulation results, oscillation stops at about 900 µA at the slow corner, while it stops at about 1.2 mA at the typical corner and at about 1.5 mA at the fast corner. On another side, the saturated output current of the ECBCM array block, *i*_outsat_, varies from around 650 µA to around 1.15 mA in the range of slow-corner devices to fast-corners. As aforementioned, if *i*_ECBCM,max_ > *I*_CCO,max_, the pattern will be step-like, and if *i*_ECBCM,max_ < *I*_CCO,max_, the pattern will show a decreasing trend. Both cases are possible for each compartment of different chips.

**Figure 7:**
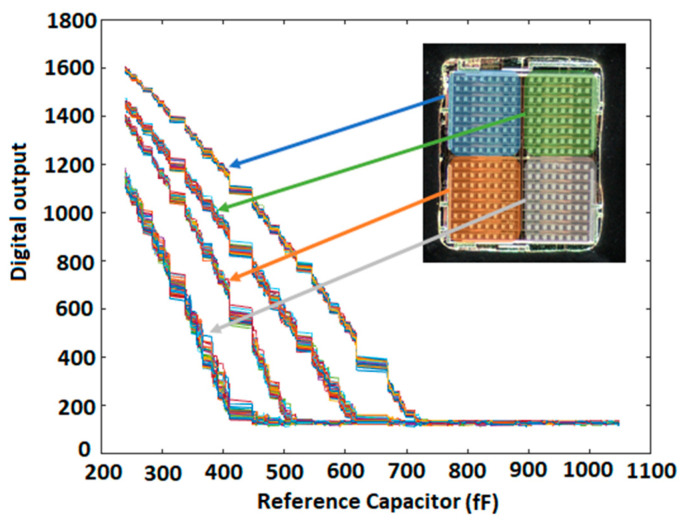
Experimental results of a chip with decreasing pattern for both compartments without sample: digital output versus different values of reference capacitor for four different electrodes in four different lobes of the array when there is no sample on the sensing area. The observed variation can be attributed to the effects of process variations on the upper compartments (highlighted in blue and green) and lower compartments (highlighted in orange and grey), as well as the influence of passivation layers atop the electrodes (highlighted in blue and orange areas).

As shown in Figure 5, the set of curves obtained for the passivated IDEs has a slightly higher capacitance compared to the non-passivated IDEs. In the step patterns shown in Figure 6, the sharp drop edge happens for higher *C*_R_ capacitances for passivated IDEs than for non-passivated IDEs. This also shows that the passivated IDEs have a higher capacitance than the non-passivated IDEs. Figure 7 illustrates the characterization results of a chip whose both compartments have decreasing patterns. This figure shows the results for all 256 electrodes of the array. As can be seen in the figure, increasing the value of the reference capacitor results in lower digital outputs because the output is proportional to ∆*C* = *C*_S_ − *C*_R_. The decreasing pattern can be seen for all electrodes. However, process variation affects the values achieved for the two upper and lower compartments of the array. Moreover, the values obtained for the passivated electrodes are slightly higher than the non-passivated ones. The measurement results discussed in the rest of the paper have been obtained using this chip.

### 5.2. Monitoring the Shape and Location of the Droplets

**Figure 8:**
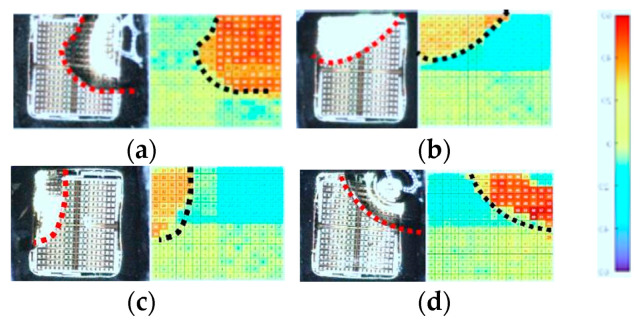
The microscopic images of the array chip during the droplet test (on the left side) and the differential values of the output with respect to the baseline (on the right side) by sweeping all reference electrodes and all sensing electrodes while the top of the sensing area is covered by a lid: (**a**) 0.5 µL pure water, (**b**) 0.5 µL of 20% ethanol in pure water, (**c**) 0.5 µL of 60% ethanol in water, and (**d**) 0.5 µL of 20% methanol in water.

Various droplets were introduced to the array sensor. Figure 8a–d is obtained for 0.5 µL pure water, 0.5 µL of 20% ethanol in pure water, 0.5 µL of 60% ethanol in water, and 0.5 µL of 20% methanol in water, respectively. The metric used in these maps is the differential value obtained from the subtraction of the measured output in the presence of a droplet above the chip from the baseline, as seen in Figure 8a–d. Since each full-page scanning using the array chip takes about 7 min, the top of the sensing area was covered by a lid to decrease the ToE of the droplet and provide enough time for full-page scanning. Then, the droplet footprint was mapped which can be matched with the captured image with acceptable accuracy. The goal of this experiment is to show the ability of the sensor to discriminate the covered and uncovered area in the sample (which are highlighted by dotted lines in Figure 8a–d). As seen in these figures, each pixel under the droplet shows a higher differential value in comparison to the uncovered ones.

#### 5.2.1. Dielectric Sensing

**Figure 9:**
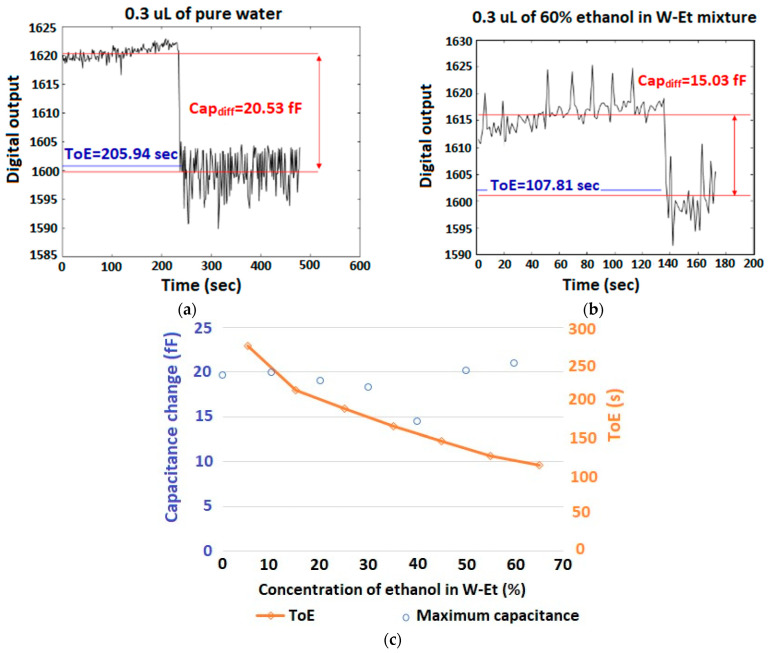
The curves of capacitance versus time with the smallest *C*_R_ for 0.3 µL of (**a**) pure water, (**b**) 60% ethanol in W-Et mixture, and (**c**) variations of ToE and maximum capacitance for 0% to 60% of ethanol in W-Et mixture.

Figure 9a,b illustrates the curves of capacitance versus time for 0.3 µL of pure water and 60% ethanol in a W-Et mixture, respectively. Figure 9c illustrates the variations of the ToE and maximum capacitance for different concentrations of 0% to 60% ethanol in the W-Et mixture. For concentrations of more than 60% ethanol or methanol in W-Et and W-Mt mixtures, the liquid does not form a droplet shape and is dispensed over the sensing area. As a result, the capillary effect of the chamber walls affects the volume on top of the sensing area and, consequently, the ToE of the sample. As seen in these figures, in this range of ethanol concentrations, the capacitance change during the saturation region is less than the ToE variations.

To calculate the values of *CCR* and *TCR*, we can use Equations (1) and (2):

(1) $$CCR=\frac{1}{100}\sum_{i=0}^{5}\frac{C_{10i}-C_{10(i+1)}}{C_{10i}}$$

(2) $$TCR=\frac{1}{100}\sum_{i=0}^{5}\frac{{ToE}_{10i}-{ToE}_{10(i+1)}}{{ToE}_{10i}}$$

where the indexes of *C* and the ToE denote the concentration of liquid 2 in liquid 1 at which the values of capacitance and ToE are respectively measured. Using Equations (1) and (2), the *CCR* and *TCR* of the experimental results shown in Figure 9c will be, respectively, *CCR* = 2.576734% and *TCR* = −13.4246%. As abovementioned when the liquid layer on top of the sensor is thicker than the *SL* (τ≫Sl), the capacitance variation is not visible by the sensor due to saturation and, as predicted in Section 3.1, *TCR* >> *CCR*. It is worth mentioning that the capacitance value is error-prone due to the non-idealities of the system which are discussed in Section 6.4.

#### 5.2.2. Time of Evaporation Measurement

**Figure 10:**
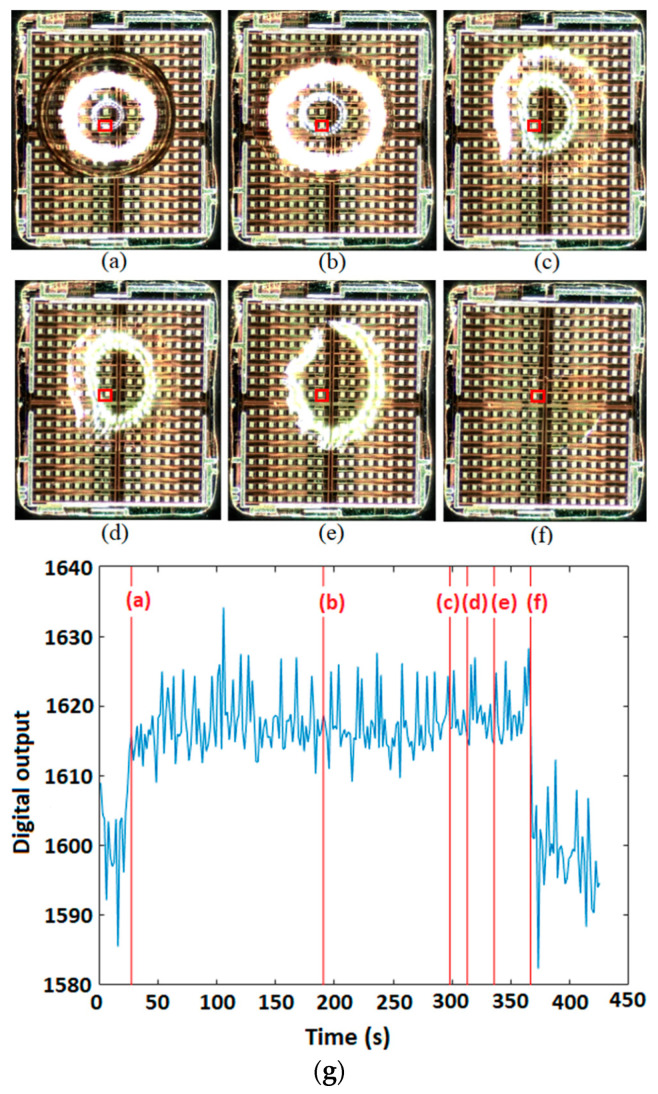
(**a**–**f**) Six screenshots of a 0.3 µL water droplet on the chip being evaporated and (**g**) the corresponding points in the capacitance–time curve obtained by the IDE at the 8th row and 8th column of the array (shown by a red rectangle in the images).

Figure 10a–f illustrates six microscopic snapshots of a 0.3 µL water droplet on top of the sensing area. The related capacitance–time curve and the corresponding points are also shown in Figure 10g. As seen in these figures, the capacitive sensor can detect the presence and absence of the droplet on top of the chip.

**Figure 11:**
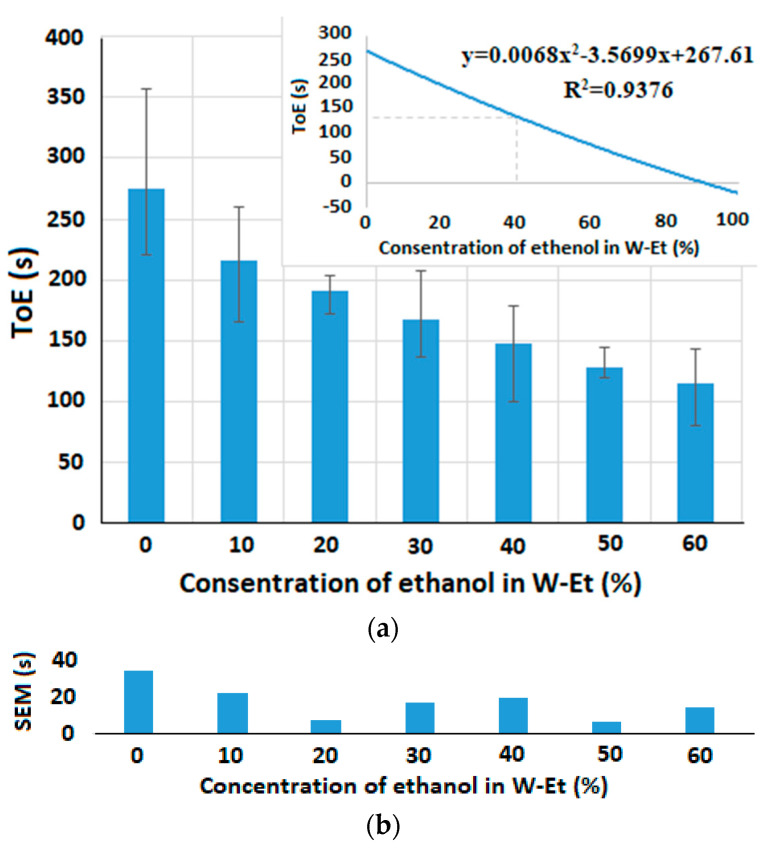
ToE for 0.3 µL of (**a**) different concentrations of ethanol in W-Et mixture, and (**b**) SEMs of the results shown in (**a**) for different concentrations of ethanol (*REC_max_* = 21.28463).

**Figure 12:**
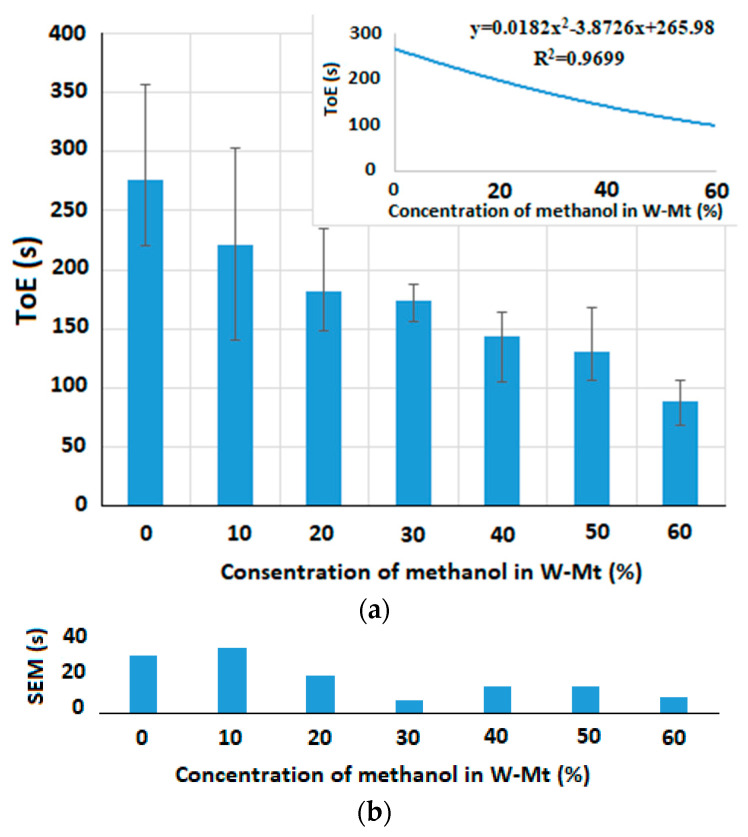
Time of evaporation for 0.3 µL of (**a**) different concentrations of methanol in W-Mt mixture, and (**b**) SEMs of the results shown in (**a**) for different concentrations of ethanol (*REC_max_* = 20.60952).

Other experiments were conducted to observe the relationship between the ToE and various concentrations of the added alcohol to the water–alcohol mixtures. Figure 11a and Figure 12a illustrate the ToE for 0.3 µL of different concentrations of, respectively, ethanol and methanol in W-Et and W-Mt mixtures at room temperature. A polynomial curve is also fitted to the results which can help to measure the concentration of alcohol (or horizontal axis) in the mixtures based on the obtained ToE (or vertical axis).

The array capacitive sensor scans the electrodes one by one and its speed is not enough to simultaneously measure the ToE of the sample on top of the all electrodes. So, we use one of the electrodes in each experiment to measure the ToE. Here, we selected one of the electrodes in the center of the chip (at the 8th row and 8th column) to avoid the capillary effects of the walls. The measurements by this electrode were repeated until the droplet was completely evaporated and all values of the reference capacitors were swept in each repetition. The sensing area was not covered with any lid. As seen in Figure 11a and Figure 12a, the ToE of the sample decreases with any increase in the concentration of the two types of alcohol in the sample.

Figure 11b and Figure 12b show the standard deviation of the mean (*SEM*) for different concentrations of liquid 2 in liquid 1. This factor is defined as $SEM=S/\sqrt{n}$, where *S* denotes the standard deviation and *n* stands for the number of observations which is equal to three (*n* = 3) in these experiments. Here, we define another parameter called the maximum relative error of concentration (*REC_max_*) to estimate the resolution of the concentration measurement in the range of 0% to 100% variations in the concentration of liquid 2 in liquid 1, which can be seen in (3):

(3) $${REC}_{max}=\frac{{SEM}_{max}}{\bar{{ToE}_{100}}-\bar{{ToE}_{0}}}\times 100$$

where *SEM_max_* stands for the maximum *SEM* obtained in different concentrations of liquid 2 in liquid 1. $\bar{{ToE}_{100}}$ and $\bar{{ToE}_{0}}$ are the average of the ToE measured for the concentrations of, respectively, 100% and 0% of liquid 2 in liquid 1. In the experimental results shown in Figure 11 and Figure 12, *REC_max_* is equal to 21.28463 and 20.60952, respectively, for W-Et and W-Mt mixtures. This means that, in the worst case, the maximum error of measuring the ethanol (or methanol) concentration in the W-Et (or W-Mt) mixture at room temperature is, in turn, 21.28463% and 20.60952% with respect to the entire range of the concentration variations.

## 6. Discussion and Future Work

There are still some practical issues that should be considered during experiments and further research is required to mitigate these non-idealities.

### 6.1. Speed of the Readout Circuit

The scanning time of all 256 electrodes (and all reference capacitors) takes about 7 min, which is higher than the required time for the evaporation of a 0.5 µL droplet. For this reason, in the experiments, we had to select one of the electrodes to measure the ToE of the sample. To increase the readout speed of the sensor, more channels are required for reading the outputs of the IDEs. Designing a CMOS capacitive sensor capable of a parallel measurement can increase the speed of measurements and help to evaluate the ToE of samples on different electrodes at the same time. If a microfluidic device is adapted to such an array structure, it will be also possible to simultaneously measure the ToE of different types of liquid.

### 6.2. Mismatches

To achieve a single pattern in all fabricated chips, the output maximum current range of the core-CBCM capacitance-to-current converter must be sufficiently lower than the input current range of the connected CCO with enough margins. Moreover, the two compartments can be merged into a 16 × 16 array of electrodes. Furthermore, here we have used two types of electrodes, passivated and non-passivated, to get an assessment of the effect of a passivation layer on the measurements. It is possible to use a single type of electrode in all pixels of the array.

### 6.3. Interferences

Implementing several IDEs in an array structure can cause cross-talk effects and increase the interferences of the system. These issues must be analyzed and mitigated in the electronic system.

### 6.4. Manual Pipetting Errors

Different factors can affect the accuracy of pipetting. For example, variation in depth during aspiration can change the volume of the sample [45]. Furthermore, liquids with a higher density (e.g., dichloromethane) have a greater mass per unit volume and impose more gravitational force on the air space between the piston and liquid. Increased air space causes a smaller volume of liquid to be aspirated into the tip [46]. The viscosity of the liquid, or in other words, the liquid’s resistance to flow is another factor affecting the accuracy of the ToE measurement using pipetting because it determines how fast or slow the liquid flows when aspirating and dispensing by the pipette [47]. If any errors occur during pipetting, it can lead to sample volume inconsistencies. Moreover, if pipettes and liquids do not equilibrate to ambient temperature or the temperature is not within the thresholds defined by the manufacturer’s precision specification, random errors occur which will prevent achieving reproducible results [48]. So, the pipettes should be selected appropriately based on the specific properties of each type of liquid and also employed correctly.

### 6.5. The Non-Flat Sensing Area Inside the Chamber

The sensor surface is not flat due to the IDEs, so the evaporation pattern of the liquid droplet in the hole can differ in comparison to a plain surface. Furthermore, the capillary impact of the wall of the chamber might affect the sample volume introduced to the electrodes.

### 6.6. Misalignment of the Electrode and Droplet

The sensed volume of the sample can be affected by the appropriate location of the droplet on top of the sensing electrodes and the electrode coverage, like when some amount of volume covers the non-sensitive areas, such as the area between or around the electrodes. Providing a microfluidic device can help to control the sample and put it more precisely on top of the sensing surface. Furthermore, designing a new chip, including the sensing electrodes that cover the whole bottom of the chamber, can help to avoid wasting the volume.

### 6.7. Bubble Creation

Another phenomenon that has been observed during the experiments is bubble creation which can result in meaningless patterns in the output, fluctuating based on the presence or absence of the bubble. A microfluidic device can also help to control this phenomenon.

### 6.8. Controlling the Environmental Effects

The temperature gradient and the humidity around the sensing area and inside the box can also cause errors in this system which are due to the non-ideal incubation of the platform, as well as the non-ideality of the hot plate and the thermocouple for adjusting the temperature. These errors can be decreased by providing a local temperature and humidity sensor, as well as a heater on top of the chip and also better incubating the platform.

### 6.9. Complexity of the Fluidic Sample

The phenomena happening on the surface are strongly dependent on the type of liquid introduced to the sensing area. In this paper, water–alcohol mixtures were used as the sample which causes reversible physical phenomena on the surface. Nonetheless, in more complex liquids, if chemical reactions occur between the molecules of liquid 1 and liquid 2, the relation between the ToE and the concentration of each liquid will be more complicated due to their sophisticated response. A study of the relationship between the ToE of more complex liquids such as wine, blood, etc., and the concentration of alcohol in these liquids can open a new avenue to develop the applications of the proposed ToE measurement technique.

### 6.10. Reliability

**Figure 13:**
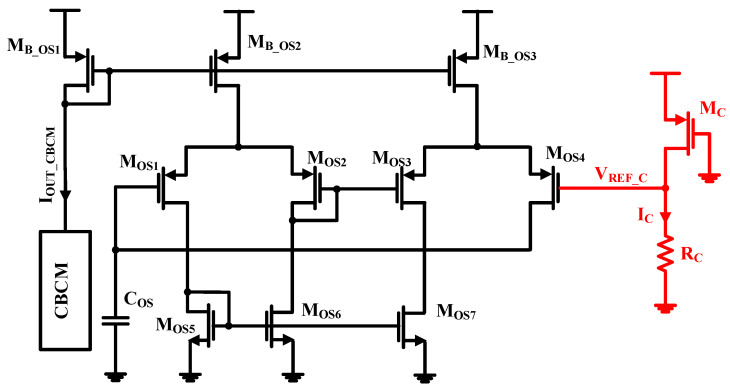
The oscillation circuit with adaptive biasing circuit.

The reliability issues in MOSFET transistors result from a high electric field causing the threshold voltage (*V*_th_) to increase and mobility (*μ*) degradation [49,50,51]. These effects are uncontested and have significant deleterious effects on the circuit performance, especially in oscillator circuits. Various strategies have been introduced to address this issue, such as over-designing circuits, with a resultant increase in the power dissipation and chip area. This paper investigates an adaptive biasing circuit to improve the reliability and variability of the oscillator. The adaptive biasing circuit provides resilience performance to process variability and reliability variation through the threshold voltage adjustment of the oscillator’s transistors. As a result the oscillation frequency variation will be diminished. Figure 13 shows the oscillator with an adaptive biasing circuit (the red par). When the threshold voltage increases due to the reliability issue, the output current of CBCM (*I*_OUT_CBCM_) will be decreased; on the other hand, according to Equation (4), the current *I*_C_ will decrease which leads to the decrease in the voltage *V*_REF_C_ (see Equation (5)). As a result, according to Equation (6), the compensation process arising from reliability degradation will be done.

(4) $$I_{C}\approx \frac{1}{2}\;\mu \;Cox\;{\left(\frac{W}{L}\right)}_{C}\;{\left(V_{SG}-V_{thC}\right)}^{2}\approx \frac{\beta_{C}}{2}{\left(V_{DD}-V_{thC}\right)}^{2}$$

(5) $$V_{REF\_C}={R_{C}I}_{C}=\frac{\beta_{C}}{2}{\;R}_{C}\;{\left(V_{DD}-V_{thC}\right)}^{2}$$

(6) $$f_{OSC}=\frac{I_{OUT\_CBCM}}{2\;C\;V_{REF\_C}}$$

### 6.11. Droplet on Chip

The goal of this paper is not the measurement of the evaporation rate; rather, the role of the capacitive sensor with the planar electrodes is to detect the presence of the droplet and estimate the total evaporation time. Therefore, the capacitor can saturate when the droplet volume exceeds a specified threshold. However, the capacitive sensor has not been designed to detect capacitance changes in the saturation mode. When the droplet size exceeds the screen length, the changes in capacitance become imperceptible. Nonetheless, the capacitive sensor can still detect the presence of liquid on the surface and infer the shape of the droplet. Thus, the total evaporation time can be estimated based on the duration of liquid presence on the sensing surface.

### 6.12. Noise

**Figure 14:**
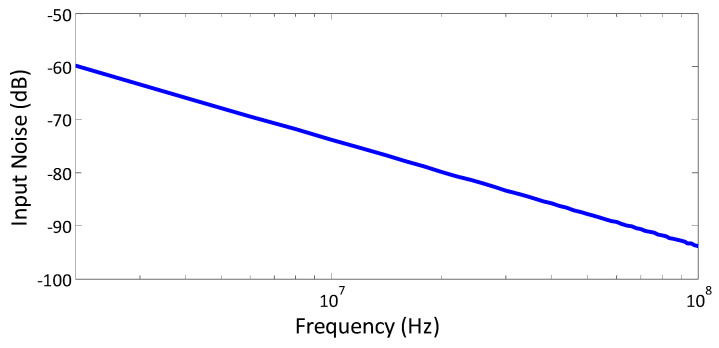
The input noise of CCO.

The effect of noise on the oscillator or other building blocks in this CMOS chip may slightly alter the capacitance measurement. However, in this paper, the CMOS capacitive sensor is utilized to detect the presence of liquid on the chip and consequently measure the ToE. Therefore, considering the measured ToE falls within a range of several minutes, the error introduced by noise might not significantly impact the measured ToE. On the other hand, the proposed sensing mechanism demonstrates noise robustness. The input noise verse frequency of CCO is depicted in Figure 14.

## 7. Conclusions

In this paper, we introduce a novel approach to developing a CMOS capacitive sensing platform for the analysis of droplet mixtures of water and alcohol. A new sensing mechanism is presented and its functionality validated using a CMOS capacitive sensor array exposed to chemical solvents with varying total ToE. We provide a theoretical analysis related to the capacitive sensor due to the change of dielectric. Building upon this theory, we demonstrate the alignment between empirical and theoretical aspects. However, our goal in this paper does not involve extending the discussion to non-electronic concepts, such as those associated with evaporation theory, etc. We discuss the measurement results, including their non-idealities, and propose potential solutions for consideration in future designs. The circuit methodology and experimental outcomes successfully highlight the advantages of CMOS capacitive sensors for alcohol–water droplet analysis with potential applications in biotechnology and the pharmaceutical industry. It is noteworthy that our focus in this paper has been on analyzing non-conductive solutions, and the experiments have not been extended to include ionic solutions. Therefore, the paper and discussions have been developed around this primary objective. However, the use of the droplet method for ionic solutions is entirely feasible, and we intend to conduct related research to explore the advantages of this method for other types of solutions, including ions.

## Data Availability

Data is contained within the article.
